# Remote Patient Management May Reduce All-Cause Mortality in Patients With Heart-Failure and Renal Impairment

**DOI:** 10.3389/fmed.2022.917466

**Published:** 2022-07-11

**Authors:** Marcel G. Naik, Klemens Budde, Kerstin Koehler, Eik Vettorazzi, Mareen Pigorsch, Otto Arkossy, Stefano Stuard, Wiebke Duettmann, Friedrich Koehler, Sebastian Winkler

**Affiliations:** ^1^Charité—Universitätsmedizin Berlin, Department of Nephrology and Medical Intensive Care, Charité University Medicine Berlin, Berlin, Germany; ^2^Berlin Institute of Health, Charité Medical University of Berlin, Berlin, Germany; ^3^Charité—Universitätsmedizin Berlin, Medical Department, Division of Cardiology and Angiology, Centre for Cardiovascular Telemedicine, Berlin, Germany; ^4^University Medical Center Hamburg-Eppendorf, Institute of Medical Biometry and Epidemiology, Hamburg, Germany; ^5^Charité—Universitätsmedizin Berlin, Institute of Biometry and Clinical Epidemiology, Berlin, Germany; ^6^Global Medical Office, Clinical and Therapeutical Governance Europe Middle East Asia, Fresenius Medical Care, Bad Homburg, Germany; ^7^German Center for Cardiovascular Research (DZHK), Gottingen, Germany; ^8^Unfallkrankenhaus Berlin, Department of Internal Medicine, Berlin, Germany

**Keywords:** remote patient management, cardiovascular disease, chronic kidney disease, heart failure, randomized controlled trial

## Abstract

**Background:**

Remote patient management (RPM) in heart failure (HF) patients has been investigated in several prospective randomized trials. The Telemedical Interventional Management in Heart Failure II (TIM-HF2)-trial showed reduced all-cause mortality and hospitalizations in heart failure (HF) patients using remote patient management (RPM) vs. usual care (UC). We report the trial's results for prespecified eGFR-subgroups.

**Methods:**

TIM-HF2 was a prospective, randomized, controlled, parallel-group, unmasked (with randomization concealment), multicenter trial. A total of 1,538 patients with stable HF were enrolled in Germany from 2013 to 2017 and randomized to RPM (+UC) or UC. Using CKD-EPI-formula at baseline, prespecified subgroups were defined. In RPM, patients transmitted their vital parameters daily. The telemedical center reviewed and co-operated with the patient's General Practitioner (GP) and cardiologist. In UC, patients were treated by their GPs or cardiologist applying the current guidelines for HF management and treatment. The primary endpoint was the percentage of days lost due to unplanned cardiovascular hospitalizations or death, secondary outcomes included hospitalizations, all-cause, and cardiovascular mortality.

**Results:**

Our sub analysis showed no difference between RPM and UC in both eGFR-subgroups for the primary endpoint (<60 ml/min/1.73 m^2^: 40.9% vs. 43.6%, *p* = 0.1, ≥60 ml/min/1.73 m^2^ 26.5 vs. 29.3%, *p* = 0.36). In patients with eGFR < 60 ml/min/1.73 m^2^, 1-year-survival was higher in RPM than UC (89.4 vs. 84.6%, *p* = 0.02) with an incident rate ratio (IRR) 0.67 (*p* = 0.03). In the recurrent event analysis, HF hospitalizations and all-cause death were lower in RPM than UC in both eGFR-subgroups (<60 ml/min/1.73 m^2^: IRR 0.70, *p* = 0.02; ≥60 ml/min/1.73 m^2^: IRR 0.64, *p* = 0.04). In a cox regression analysis, age, NT-pro BNP, eGFR, and BMI were associated with all-cause mortality.

**Conclusion:**

RPM may reduce all-cause mortality and HF hospitalizations in patients with HF and eGFR < 60 ml/min/1.73 m^2^. HF hospitalizations and all-cause death were lower in RPM in both eGFR-subgroups in the recurrent event analysis. Further studies are needed to investigate and confirm this finding.

## Introduction

Heart failure (HF) is a highly prevalent syndrome associated with increased mortality, morbidity, and detrimental effect on the quality of life (1). Due to demographic changes number of patients with HFare expected to increase further (2). There is a strong interrelation between the heart and kidneys implying that the declining function of one affects the function of the other, resulting in cardiorenal syndrome (3–6). Chronic kidney disease (CKD) is an important independent risk factor for poor outcomes in patients with cardiovascular disease (7). CKD is a comorbidity in up to 12% of patients with HF (8, 9). In patients with the cardiorenal syndrome, congestive decompensation is particularly common and entails hospitalization in many cases (3). In patients hospitalized with HF, the fraction of patients with renal impairment increases up to 40% (1, 10, 11). The extent of re-hospitalizations and length of hospital stay raises with advanced CKD stages (12).

Diuretics are highly effective in the treatment of hydropic decompensation. But the response to diuretics depends on renal function and the user has an impact on the risk of acute kidney injury and recurrent hydropic decompensation (13, 14). Because of the close interaction between HF and renal function with the need for close monitoring and therapy modification, telemedical interventions in higher CKD stages may offer benefits (14–17).

Despite guideline-directed medical treatment, device therapy and interventional therapies (coronary revascularization, heart valve repair, cardiac implants) patients with HF are at risk for disease progression and acute worsening. Remote patient management (RPM) is a new structured care approach combining telemonitoring of vital signs, information from sensors, and cardiac implants with educational support and collaborative care in a heart failure network. Early detection of worsening followed by early intervention with therapy adjustment creates a tight outpatient feedback loop to avoid hospitalization. RPM has been shown to stabilize the patient's condition and achieve better outcomes, particularly after hospitalization for worsening HF. This has been demonstrated for invasive and non-invasive telemonitoring (18, 19).

The Telemedical Interventional Management in Heart Failure II (TIM-HF2) trial investigated remote patient care in patients with HF (20, 21). Like in other trials RPM was associated with a significant reduction in the percentage of days lost due to unplanned cardiovascular hospital admissions and all-cause death (18, 20, 22–26). At the study entry, the trial stratified patients according to their eGFR, and the protocol prespecified subgroup analysis of patients with renal impairment. The aim of this study was to analyze the effect of RPM on hospitalizations and mortality in HF patients with impaired (eGFR < 60 ml/min/1.73 m^2^) vs. better renal function (eGFR ≥60 ml/min/1.73 m^2^) and to analyze the impact of renal function on the study outcomes.

## Methods

### Study Design

The TIM-HF2 trial, registered at clinicalTrials.gov (NCT01878630), was a prospective, randomized, controlled, parallel-group, unmasked (with randomization concealment), and multicenter trial with pragmatic elements introduced for data collection. Detailed methods and overall results of this trial have been published (20, 21).

### Setting and Participants

The trial was performed in Germany from 2013 to 2017. Inclusion criteria were stable heart failure in New York Heart Association functional class II or III, hospital admission due to worsening heart failure within 12 months before randomization, a guideline-directed medical therapy with left ventricular function (LVEF) ≤45%, or being on diuretics with LVEF ≥ 45%. Key exclusion criteria were end-stage renal disease, major depression (Depression model of the Patient Health Questionnaire-9 score >9), recent hospitalization (7 days for any cause, 1 month before cardiac interventions like coronary revascularization or cardiac resynchronization therapy implantation), or planned cardiac procedures.

Patients were randomly assigned (1:1) to either RPM including usual care (UC) or UC alone (21). Renal function was determined by creatinine measurements at baseline and estimation of glomerular filtration rate using the CKD-EPI formula. Subgroups were prespecified into three strata of renal function (<30, 30–60, ≥ 60 ml/min/1.73 m^2^) based on eGFR at baseline (21). As groups of eGFR < 30 ml/min/1.73 m^2^ were small in both arms, we merged them with the eGFR-group 30–60 ml/min/1.73 m^2^ for further analysis. Important clinical variables were balanced using Pocock's minimization algorithm at randomization (21). No interim analyses were planned.

### Intervention

The RPM intervention consisted of a daily review of transmitted vital parameters (bodyweight, blood pressure, electrocardiogram, peripheral capillary oxygen saturation, and self-rated health status on a scale) from the patient's home to the telemedical center (TC); the patient education; and co-operation between the TC, and the patient's General Practitioner (GP) and cardiologist. Following standard operating procedures an individualized assessment of the clinical status, medication, and adherence of the patients was done daily. In the case of need, therapy adjustments were recommended to the GP or instituted directly to the patient by the TC physician outside business hours. There was 24/7 physician-guided emergency support by the TC. On a monthly basis, the HF nurses performed structured telephone interviews via telephone.

Patients allocated to UC were followed up in accordance with the current guidelines for HF management and treatment (27). Throughout the study follow-up, the patient's GP and cardiologist were free to adjust or prescribe treatments in accordance with the patient's clinical condition.

Patients in both study groups were followed up for at least 365 days and up to 393 days after randomization. All patients were examined by their cardiologists at the screening and baseline visit and at the final study visit; the latter was done on day 365 (28-day time window) after randomization. In between, patient visits were undertaken by the patient's GP or local cardiologist scheduled at 3, 6, and 9 months. At all visits, data were collected in a case report form, which included vital signs and body weight, and the patients were asked about the occurrence of hospital admissions since the last study contact. A crosscheck with health insurance companies was done to avoid collection bias and ensure complete hospitalization data. The study was conducted along with the Principles in the Declaration of Helsinki and the laws and regulations in Germany. All participants gave their informed consent. Ethics approval was obtained from the local ethics committee.

### Outcomes

The primary endpoint was the percentage of days lost due to unplanned cardiovascular hospitalization or death from any cause, comparing RPM with UC during the individual patient follow-up time. The main secondary outcomes were all-cause mortality and cardiovascular mortality during the individual patient follow-up time plus 28 days after the last study visit, to a maximum of 393 days; percentage of days lost due to unplanned cardiovascular hospitalizations, and percentage of days lost due to unplanned heart failure hospitalizations.

### Statistical Analysis

We used IBM SPSS Statistics, Version 27, and R version 4.1.0 for the analysis of the data (28, 29). All analyses were performed according to the intention to treat principles on the full analysis set. Laboratory data were imputed using last-observation-carried-forward in case the patient survived. The highest amount of missingness in a single laboratory value was less than 6%. To categorize the patients according to their kidney function at baseline, the eGFR was calculated using the CKD-EPI-formula to define the subgroups (30). All analyses of this study are secondary and therefore exploratory. The following analyses are done separately for the subgroups regarding eGFR. The main study's primary endpoint percentage of days lost due to unplanned cardiovascular hospitalization or death from any cause was compared as a weighted average between the randomized groups using a permutation test using individual observation time as weights. Kaplan-Meier analysis was applied for the combined endpoint of all-cause mortality and first rehospitalization. The potentially recurrent events unplanned cardiovascular hospitalizations and unplanned heart failure hospitalizations were analyzed with the use of negative binomial models on the number of distinct events per patient accounting for all-cause or cardiovascular death as terminal event and incidence rate ratios (IRR) were calculated. Recurrent event analyses were performed for the combined endpoints unplanned cardiovascular hospitalizations and cardiovascular mortality, unplanned cardiovascular hospitalizations and all-cause mortality, unplanned heart failure hospitalizations and cardiovascular mortality, and unplanned heart failure hospitalizations and all-cause mortality. A Cox regression analysis was performed to investigate the influence of covariates for all-cause death. Covariates included demographic parameters at randomization (sex, age, BMI), and laboratory or diagnostic findings at baseline (sodium, potassium, NTproBNP, LVEF). As the analysis was performed for the whole cohort, the prespecified eGFR subgroup and randomization arm were included too. An interaction between eGFR and sodium at baseline was investigated.

## Results

**Figure 1:**
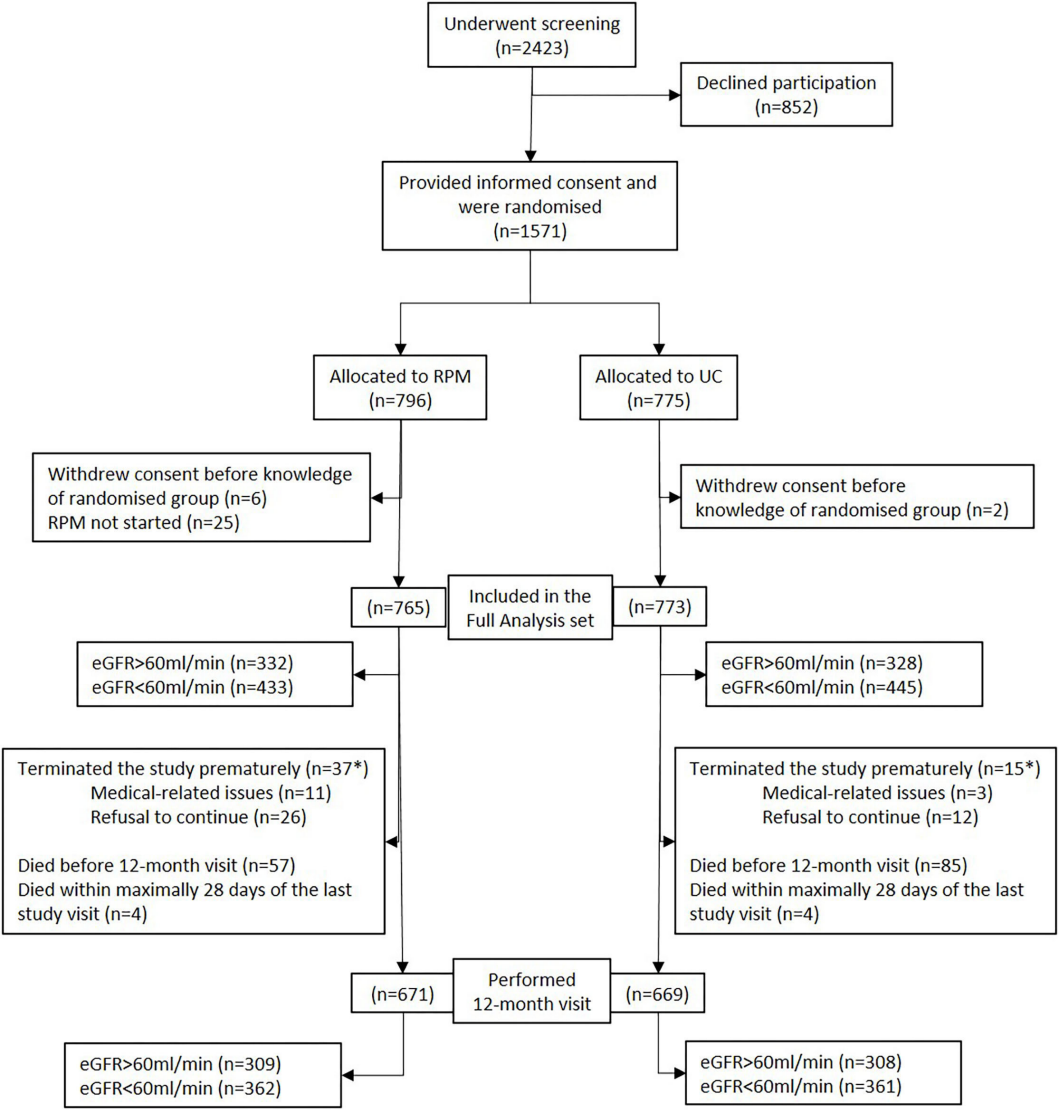
Flowchart of the cohort. eGFR, estimated glomerular filtration rate; RPM, remote patient management; UC, usual care. *Survival status known up to 393 days post randomization for all patients who withdrew prematurely.

Overall, 1,571 patients were included in the study (Figure 1). Allocated into the RPM group were 796 patients, of whom 765 (96.1%) were included in the full analysis set. Allocated into usual care (UC) were 775 patients of whom 773 (99.7%) were included in the full analysis set. The 12-month-visit was performed in 671 (87.7%) in RPM and 669 (86.5%) in UC.

Table 1 shows baseline data of patients in both study arms regarding their kidney function.

**Table 1:** Demographics of remote patient management (RPM) and control group (UC) with regard to eGFR at randomization.

	**eGFR < 60 ml/min/1.73 m^2^**		**eGFR ≥60 ml/min/1.73 m^2^**	
**Group**	**RPM**	**UC**	**RPM**	**UC**
N	433	445	332	328
Age (years)	74 ± 8	74 ± 9	65 ± 11	66 ± 11
Sex (male)	283 (65.4%)	288 (64.7%)	250 (75.3%)	249 (75.9%)
Living alone (%)	113 (26.1%)	125 (26.1%)	100 (30.1%)	97 (29.6%)
Living in a city area vs. rural area				
Rural	260 (60.0%)	249 (56.0%)	197 (59.3%)	209 (63.7%)
Urban	173 (40.0%)	196 (44.0%)	135 (40.7%)	119 (36.3%)
NYHA at Baseline				
I	0	1 (0.2%)	3 (0.9%)	7 (2.1%)
II	197 (45.5%)	199 (44.7%)	203 (61.1%)	197 (60.1%)
III	234 (54.0%)	244 (54.8%)	125 (37.7%)	123 (37.5%)
IV	2 (0.5%)	1 (0.2%)	1 (0.3%)	1 (0.3%)
Left ventricular ejection fraction according to categorization to the study protocol				
≤ 45%	275 (63.5%)	299 (67.2%)	217 (65.4%)	210 (64%)
>45%	158 (36.5%)	146 (32.8%)	115 (34.6%)	118 (36%)
Left ventricular ejection fraction according to categorization to current ESC guidelines *post-hoc*				
HFrEF ≤ 40%	230 (53.1%)	236 (53.0%)	179 (53.9%)	173 (52.7%)
HFmrEF 40–49%	56 (12.9%)	74 (16.6%)	46 (13.9%)	48 (14.6%)
HFpEF ≥ 50%	147 (34.0%)	135 (30.3%)	107 (32.2%)	107 (32.6%)
Days between discharge of last heart failure hospitalization and randomization				
≤ 30 days	118 (27.3%)	117 (26.4%)	80 (24.1%)	75 (22.9%)
31–90 days	155 (35.8%)	150 (33.8%)	121 (36.4%)	131 (39.9%)
>90 days	160 (37.0%)	177 (39.9%)	131 (39.5%)	122 (37.2%)
Weight at randomization (kg)	83 (72–97)	83 (72–96)	87 (76–103)	88 (77–102)
BMI (kg/m^2^)	28.1 (24.5–33.1)	28.7 (24.9–33.2)	29.3 (25.7–33.6)	29.3 (25.9–33.6)
Blood pressure (mmHg)				
Systolic	125 (110–140)	120 (110–140)	125 (110–140)	123 (110–136)
Diasystolic	70 (65–80)	70 (65–80)	77.5 (70–80)	75 (69–80)
Pulse (beats/min)	71 (62–81)	70 (61–79)	70 (62–80)	72 (61–82)
**Primary cause of heart failure**				
Ischemic cause (coronary artery disease/myocardial infarction)	198 (45.7%)	207 (46.5%)	103 (31%)	116 (35.4%)
Hypertension	75 (17.3%)	85 (19.1%)	53 (16%)	61 (18.6%)
Dilated cardiomyopathy	67 (15.5%)	84 (18.9%)	109 (32.8%)	87 (26.5%)
Other	93 (21.5%)	69 (15.5%)	67 (20.2%)	64 (19.5%)
**Cardiovascular risk factors**				
Smoking				
Unknown	9 (2.1%)	17 (3.8%)	15 (4.5%)	10 (3.0%)
Non-smoker	240 (55.4%)	233 (52.4%)	138 (41.6%)	152 (46.3%)
Former smoker	157 (36.3%)	174 (39.1%)	129 (38.9%)	130 (39.6%)
Smoker	27 (6.2%)	21 (4.7%)	50 (15.1%)	36 (11.0%)
Hyperlipidemia				
Unknown	25 (5.8%)	21 (4.7%)	16 (4.8%)	18 (5.5%)
No	149 (34.4%)	162 (36.4%)	157 (47.3%)	156 (47.6%)
Yes	259 (59.8%)	262 (58.9%)	159 (47.9%)	153 (46.6%)
Diabetes mellitus	218 (50.3%)	222 (49.9%)	129 (38.9%)	133 (40.5%)
**Medical history**				
Coronary revascularization (PCI)	172 (39.7%)	187 (42.0%)	90 (27.1%)	111 (33.8%)
Coronary artery bypass surgery	87 (20.1%)	94 (21.1%)	47 (14.2%)	51 (15.5%)
TAVI	15 (3.5%)	28 (6.3%)	7 (2.1%)	2 (0.6%)
Mitral-Clip	21 (4.8%)	26 (5.8%)	5 (1.5%)	8 (2.4%)
Cardiac surgery for valves	48 (11.1%)	41 (9.2%)	38 (11.4%)	30 (9.1%)
Implantable cardioverter defibrillator (ICD)	134 (30.9%)	154 (34.6%)	90 (27.1%)	80 (24.4%)
Cardiac resynchronization therapy (CRT)	81 (18.7%)	95 (21.3%)	37 (11.1%)	27 (8.2%)
Ablation of pulmonary veins	38 (8.8%)	32 (7.2%)	33 (9.9%)	20 (6.1%)
**Laboratory measurements**				
Hemoglobin (g/dl)	12.9 (11.4–14.0)	12.9 (11.7–14)	13.7 (12.6–14.8)	13.8 (12.8–14.9)
Serum sodium (mmol/l)	140 (137–141)	140 (138–142)	139 (138–142)	140 (138–142)
Serum potassium (mmol/l)	4.5 (4.2–5.0)	4.6 (4.2–5.0)	4.5 (4.2–4.8)	4.4 (4.1–4.7)
Serum creatinine (mg/dl)	1.5 (1.3–1.8)	1.5 (1.2–2.0)	1.0 (0.9–1.1)	1.0 (0.9–1.2)
eGFR CKD–EPI (ml/min/1.73 m^2^)	42 (34–51)	42 (31–51)	79 (70–90)	77 (67–88)
NT-proBNP (pg/ml)	1,947 (973–4,487)	1,943 (903–4,374)	979 (395–1,809)	1,035 (417–1,930)
Stratified by LVEF ≤ 45% vs. >45%				
NT-proBNP (pg/ml) in patients with LVEF ≤ 45%	2,328 (1,254–5,389)	2,398 (1,295–5,395)	1,107 (479–2,183)	1,118 (479–2,140)
NT-proBNP (pg/ml) in patients with LVEF >45%	1,366 (641–2,617)	1,117 (472–2,549)	703 (208–1,257)	853 (23–1,648)
Mid-regional pro-adrenomedullin (nmol/l)	1.3 (1.0–1.8)	1.3 (1.0–1.7)	0.8 (0.7–1.0)	0.9 (0.7–1.1)
**Concomitant treatment**				
ACE inhibitor or ARB	347 (80.1%)	365 (82.0%)	281 (84.6%)	276 (84.1%)
ARN inhibitor	21 (4.8%)	28 (6.3%)	23 (6.9%)	19 (5.8%)
Beta-blocker	397 (91.7%)	410 (92.1%)	305 (91.9%)	301 (91.8%)
Aldosterone antagonist	221 (51.0%)	221 (49.7%)	220 (66.3%)	184 (56.1%)
Loop diuretics	421 (97.2%)	431 (96.9%)	296 (89.2%)	290 (88.4%)
Thiazides	126 (29.1%)	119 (26.7%)	65 (19.5%)	66 (20.1%)
Other diuretics	2 (0.5%)	0 (0%)	2 (0.6%)	1 (0.3%)
Vitamin-K-antagonists	170 (39.3%)	175 (39.3%)	95 (28.6%)	97 (29.6%)
Antiplatelet therapy	63 (14.5%)	78 (17.5%)	40 (12.0%)	52 (15.9%)
DOAC	117 (27%)	117 (26.3%)	88 (26.5%)	91 (27.7%)
Platelet aggregation inhibitors	146 (33.7%)	145 (32.6%)	120 (36.1%)	122 (37.2%)
Lipid lowering	267 (61.7%)	271 (60.9%)	189 (56.9%)	182 (55.5%)
Insulin	116 (26.8%)	114 (25.6%)	54 (16.3%)	56 (17.1%)
Oral hypoglycemic	114 (26.3%)	95 (21.3%)	92 (27.7%)	90 (27.4%)
Calcium antagonist	100 (23.1%)	112 (25.2%)	63 (19%)	76 (23.2%)
Nitrate	27 (6.2%)	32 (7.2%)	10 (3%)	16 (4.9%)
Digitalis glycoside	77 (17.8%)	86 (19.3%)	42 (12.7%)	47 (14.3%)
Antiarrhythmic	72 (16.6%)	64 (14.4%)	27 (8.1%)	34 (10.4%)

*NYHA, New York heart association; HFrEF, Heart failure with reduced ejection fraction; HFmrEF, Heart failure with mildly reduced fraction; HFpEF, Heart failure with preserved ejection fraction; COPD, chronic obstructive pulmonary disease; ICD, implantable cardioverter-defibrillator; CRT, cardiac resynchronization therapy; TAVI, transcatheter aortic valve implantation; eGFR-CKDEPI, estimated glomerular filtration rate using the Chronic Kidney Disease Epidemiology Collaboration formula. Data are mean (SD) or n (%), and median (IQR) for all laboratory tests*.

In total, 878 (57.1%) had an impaired renal function (eGFR < 60 ml/min/1.73 m^2^, eGFR < 60) at randomization and 660 (42.9%) had an eGFR ≥ 60 ml/min/1.73 m^2^ (eGFR ≥ 60). The prespecified groups had 158 (10.3%) patients with eGFR < 30 ml/min/1.73 m^2^ (65 RPM and 93 UC), 720 (46.8%) patients with eGFR 30–60 ml/min/1.73 m^2^ (368 RPM and 352 UC) and 660 (42.9%), patients with eGFR ≥ 60 (332 RPM, 328 UC). As the group eGFR < 30 ml/min/1.73 m^2^ was small in both groups this group was analyzed together with 30–60 ml/min/1.73 m^2^.

Table 2 shows demographic data of all patients stratified by kidney function at baseline. In the group with impaired renal function mean eGFR was 42 ± 11 ml/min/1.73 m^2^ compared to 78 ± 13 ml/min/1.73 m^2^ in the group with better renal function. Patients with impaired renal function were older, less frequently male, had a lower BMI, a higher NT-proBNP level, and more comorbidities (2.0 ± 1.2 vs. 1.1 ± 1.0). Clinical signs of fluid overload were reported more frequently in patients with eGFR < 60. Furthermore, concomitant medication was different in patients with CKD as a number of drugs was higher in patients with eGFR < 60.

**Table 2:** Demographics of the whole cohort, impaired (eGFR < 60 ml/min/1.73 m^2^), and normal (eGFR ≥ 60 ml/min/1.73 m^2^).

	**All**	**eGFR < 60 ml/min/1.73 m^2^**	**eGFR ≥ 60 ml/min/1.73 m^2^**
*N*	1,538	878	660
Age (years)	70 ± 11	74 ± 8	66 ± 11
Sex (% male)	1,070 (69.6%)	571 (65.0%)	499 (75.6%)
Living alone (%)	435 (28.3%)	238 (27.1%)	197 (29.8%)
Living in a city are vs. rural area			
Rural	915 (59.5%)	509 (58.0%)	406 (61.5%)
Urban	623 (40.5%)	369 (42.0%)	254 (38.5%)
NYHA at Baseline (%)			
I	11 (0.7%)	1 (0.1%)	10 (1.5%)
II	796 (51.8%)	396 (45.1%)	400 (60.6%)
III	726 (47.2%)	478 (54.4%)	248 (37.6%)
IV	5 (0.3%)	3 (0.3%)	2 (0.3%)
Left ventricular ejection fraction according to categorization to the study protocol			
≤ 45%	1,001 (65.1%)	574 (65.4%)	427 (64.7%)
>45%	537 (34.9%	304 (34.6%)	233 (35.3%)
Left ventricular ejection fraction according to categorization to current ESC guidelines *post-hoc*			
HFrEF ≤ 40%	818 (53.2%)	466 (53.1%)	352 (53.5%)
HFmrEF 40–49%	224 (14.6%)	130 (14.8%)	94 (14.3%)
HFpEF ≥50%	496 (32.2%)	282 (32.1%)	214 (32.4%)
Days between discharge of last heart failure hospitalization and randomization			
≤ 30 days	390 (25.4%)	235 (26.8%)	155 (23.5%)
31–90 days	557 (36.2%)	305 (34.7%)	252 (38.2%)
>90 days	590 (38.4%)	337 (38.4%)	253 (38.3%)
Weight at randomization (kg)	87 ± 20	85 ± 19.2	90 ± 20.6
BMI (kg/m^2^)	29.7 ± 6.2	29.3 ± 6.1	30.1 ± 6.4
Blood pressure (mmHg)			
Systolic	125 ± 19.4	125 ± 19.6	126 ± 19.0
Diastolic	74 ± 11.3	73 ± 11.5	75 ± 11.0
Pulse (beats/min)	72 ± 14.0	72 ± 13.6	73 ± 14.5
**Primary cause of heart failure**			
Ischemic cause (coronary artery disease/myocardial infarction)	624 (40.6%)	405 (46.1%)	219 (33.2%)
Hypertension	274 (17.8%)	160 (18.2%)	114 (17.3%)
Dilated cardiomyopathy	347 (22.6%)	151 (17.2%)	196 (29.7%)
Other	293 (19.1%)	162 (18.5%)	131 (19.8%)
**Cardiovascular risk factors**			
Smoking			
Unknown	51 (3.3%)	26 (3.0%)	25 (3.8%)
Non-smoker	763 (49.6%)	473 (53.9%)	290 (43.9%)
Former smoker	590 (38.4%)	331 (37.7%)	259 (39.2%)
Smoker	134 (8.7%)	48 (5.5%)	86 (13.0%)
Hyperlipidemia			
Unknown	80 (5.2)	46 (5.2%)	34 (5.2%)
No	624 (40.6%)	311 (35.4%)	313 (47.5%)
Yes	833 (54.2%)	521 (59.3%)	312 (47.3%)
Diabetes mellitus (%)	702 (45.6%)	440 (50.1%)	262 (39.7%)
**Medical history**			
Coronary revascularization (PCI)	560 (36.4%)	359 (40.9%)	453 (30.5%)
Coronary artery bypass surgery	279 (18.1%)	181 (20.6%)	98 (14.8%)
TAVI	52 (3.4%)	43 (4.9%)	9 (1.4%)
Mitral-Clip	60 (3.9%)	47 (5.4%)	13 (2.0%)
Cardiac surgery for valves	157 (10.2%)	89 (10.1%)	68 (10.3%)
Implantable cardioverter defibrillator (ICD)	458 (29.8%)	288 (32.8%)	170 (25.8%)
Cardiac resynchronization therapy (CRT)	240 (15.6%)	176 (20.0%)	64 (9.7%)
Ablation of pulmonary veins	123 (8.0%)	70 (8.0%)	53 (8.0%)
Laboratory measurements			
Hemoglobin (g/dl)	13.2 (12.1–14)	12.9 (11.6–14.0)	13.8 (12.7–14.8)
Serum sodium (mmol/l)	140 (137–142)	140 (137–142)	140 (138–142)
Serum potassium (mmol/L)	4.5 (4.2–4.9)	4.8 (4.2–5.0)	4.4 (4.2–4.8)
Serum creatinine (mg/dl)	1.4 (1–1.6)	1.5 (1.2–1.9)	1 (0.9–1.1)
eGFR CKD–EPI (ml/min/1.73 m^2^)	55 (40–75)	42 (33–51)	78 (69–89)
NT-proBNP (pg/ml)	1,436 (605–3,100)	1,945 (930–4,413)	994 (414–1,884)
Stratified by LVEF ≤ 45% vs. >45%			
NT-proBNP (pg/ml) in patients with LVEF >45 (*n* = 537)	1,749 (786–3,791)	1,255 (555–2,592)	782 (239–1,489)
NT-proBNP (pg/ml) in patients with LVEF ≤ 45 (*n* = 1,001)	1,037 (416–2,030)	2,373 (1,279–5,384)	1,107 (479–2,171)
Mid-regional pro-adrenomedullin (nmol/L)	1.1 (0.8–1.5)	1.3 (1.0–1.8)	0.8 (0.7–1.0)
**Concomitant treatment**			
ACE inhibitor or ARB	1,269 (82.5%)	712 (81.1%)	557 (84.4%)
ARN inhibitor	91 (5.9%)	49 (5.6%)	42 (6.4%)
Beta-blocker	1,413 (91.9%)	807 (91.9%)	606 (91.8%)
Aldosterone antagonist	846 (55.0%)	442 (50.3%)	404 (61.2%)
Loop diuretics	1,438 (93.5%)	852 (97.0%)	586 (88.8%)
Thiazides	376 (24.4%)	245 (27.9%)	131 (19.8%)
Other diuretics	5 (0.3%)	2 (0.2%)	3 (0.5%)
Vitamin-K-antagonists	537 (34.9%)	345 (39.3%)	192 (29.1%)
Antiplatelet therapy	233 (15.1%)	141 (16.1%)	92 (13.9%)
DOAC	413 (26.9%)	234 (26.4%)	179 (27.1%)
Platelet aggregation inhibitors	533 (34.7%)	291 (33.1%)	242 (36.7%)
Lipid lowering	909 (59.1%)	538 (61.3%)	371 (56.2%)
Insulin	340 (22.1%)	230 (26.2%)	110 (16.7%)
Oral hypoglycemic	391 (25.4%)	209 (23.8%)	182 (27.6%)
Calcium antagonist	351 (22.8%)	212 (24.1%)	139 (21.1%)
Nitrate	85 (5.5%)	59 (6.7%)	26 (3.9%)
Digitalis glycoside	252 (16.4%)	163 (18.6%)	89 (13.5%)
Antiarrhythmic	197 (12.8%)	136 (15.5%)	61 (9.2%)

*Data are mean (SD) or number of patients (%), median and IQR (inter-quartile range) for laboratory measurements. ACE, angiotensin-converting enzyme; ARB, angiotensin-receptor blocker; ARN, angiotensin receptor-neprilysin; CRT, cardiac resynchronisation therapy; DOAC, direct oral anticoagulants; eGFR, estimated glomerular filtration rate using the Chronic Kidney Disease Epidemiology Collaboration formula; HFrEF, Heart failure with reduced ejection fraction; HFmrEF, Heart failure with mildly reduced fraction; HFpEF, Heart failure with preserved ejection fraction; ICD, implantable cardioverter-defibrillator; LVEF, left ventricular ejection fraction; NT-proBNP, N-terminal-pro-B-type natriuretic peptide; NYHA, New York Heart Association; TAVI, transcatheter aortic valve implantation*.

The distribution of patients with eGFR < 60 and eGFR ≥ 60 at randomization was balanced due to the randomization algorithm (RPM 56.6 and 43.4%, UC 57.6 and 42.4%).

In patients with eGFR < 60 and eGFR ≥ 60 LVEF at randomization was similar in RPM and UC as per the study protocol (≤45% vs. >45%). Applying the heart failure classification most patients showed HFrEF in both study arms.

### Study Outcomes According to Renal Function and Treatment Group

Table 3 shows the results of our analysis of the main trial's primary endpoint and key secondary outcomes comparing both study arms in patients with eGFR < 60 and eGFR ≥ 60. The effect of RPM on the primary outcome of the percentage of days lost due to unplanned cardiovascular hospitalizations or death from any cause was not significantly different in the eGFR-subgroup analysis.

**Table 3:** Outcomes comparing remote patient management (RPM) vs. usual care (UC) in patients with eGFR < 60 ml/min/1.73 m^2^ and ≥ 60 ml/min/1.73 m^2^, respectively.

	**eGFR < 60 ml/min/1.73 m** ^**2**^				**IRR RPM vs. UC**	***p*** **-value**	**eGFR ≥60 ml/min/1.73 m** ^**2**^				**IRR RPM vs. UC**	***p*** **-value**
	**RPM (*****N*** **= 433)**		**UC (*****N*** **= 445)**				**RPM (*****N*** **= 332)**		**UC (*****N*** **= 328)**			
	**Number of patients with events**	**Weighted average (95% CI)**	**Number of patients with events**	**Weighted average (95% CI)**			**Number of patients with events**	**Weighted average (95% CI)**	**Number of patients with events**	**Weighted average (95% CI)**		
Percentage of days lost due to unplanned cardiovascular hospitalizations or death from any cause	177 (40.9%)	6.7 (5.04–8.37)	194 (43.6%)	9.34 (7.44–11.23)	0.78 (0.58–1.07)	0.1	88 (26.5%)	2.56 (1.55–3.58)	96 (29.3%)	2.99 (1.89–4.09)	0.86 (0.65–1.12)	0.3
Days lost per year		24.5 days (18.4–30.5)		34.1 days (27.2–41.0)				9.3 days (5.6–13.1)		10.9 days (6.9–14.9)		
All-cause mortality	48 (11.1%)	11.24 (8.47–14.92)	74 (16.6%)	16.8 (13.37–21.09)	0.67 (0.47–0.96)	0.03	13 (3.9%)	3.75 (2.18–6.46)	15 (4.6%)	4.35 (2.62–7.22)	0.86 (0.41–1.81)	0.7
Cardiovascular mortality	30 (6.9%)	7.03 (4.91–10.05)	48 (10.8%)	10.89 (8.21–14.46)	0.64 (0.41–1.02)	0.07	9 (2.7%)	2.6 (1.35–4.99)	11 (3.4%)	3.19 (1.77–5.76)	0.81 (0.34–1.96)	0.6
Percentage of days lost due to unplanned cardiovascular hospitalizations	171 (39.5%)	2.26 (1.75–2.77)	175 (39.3%)	2.90 (2.27–3.53)	0.939 (0.71–1.23)	0.2	81 (24.4%)	1.02 (0.68–1.37)	94 (28.7%)	1.50 (1.06–1.93)	0.89 (0.74–1.07)	0.2
Days lost per year		8.3 days (6.4–10.1)		10.6 days (8.3–12.9)				3.7 days (2.5–5.0)		5.5 days (3.9–7.0)		
Percentage of days lost due to unplanned hospital admissions due to heart failure	103 (23.8%)	1.48 (1.04–1.91)	131 (29.4%)	2.06 (1.53–2.60)	0.80 % (0.63–1.02)	0.07	37 (11.4%)	0.49 (0.27–0.72)	62 (18.9%)	0.86 (0.55–1.16)	0.80 (0.69–0.95)	0.017
Days lost		5.4 days (3.8–7.0)		7.5 days (5.6–9.5)				1.8 days (1.0–2.6)		3.1 days (2.0–4.2)		

*IRR, incident rate ratio. p-value was calculated with a permutation test using individual observation time as weights*.

All-cause mortality was significantly reduced in the RPM group compared to the UC group. Overall, 61/765 patients (8.0%) in the RPM group and 89/773 (11.5%) in the UC group died (*p* = 0.02). In patients with eGFR < 60, 48/433 (11.1%) patients in the RPM group vs. 74/445 (16.6%) in the UC group died, resulting in a 33% reduction in incidence rate ratio [11.2 vs. 16.6%, IRR 0.67 (0.47–0.96), *p* = 0.03]. But this difference was not seen in patients with eGFR ≥ 60: 13/332 (3.9%) patients in the RPM group vs. 15/328 (4.6%) in the UC group died. Table 4 shows the causes of death.

**Table 4:** Reasons for Death depending on kidney function and study arm.

	**eGFR < 60 ml/min/1.73 m**^**2**^ **(*****N*** **= 878)**		**eGFR ≥60 ml/min/1.73 m**^**2**^ **(*****N*** **= 660)**		
	**RPM (*n* = 433)**	**UC (*n* = 445)**	**RPM (*n* = 332)**	**UC (*n* = 328)**	**Total**
Sudden cardiac death	8 (1.8%)	8 (1.8%)	4 (1.2%)	4 (1.2%)	24 (1.6%)
Acute decompensation of chronic heart failure	18 (4.2%)	28 (6.3%)	4 (1.2%)	4 (1.2%)	54 (3.5%)
Acute coronary syndrome/Acute myocardial infarction	1 (0.2%)	3 (0.7%)	0	2 (0.6%)	6 (0.4%)
Death due to cardiovascular surgery	2 (0.4%)	2 (0.5%)	1 (0.3%)	0	5 (0.3%)
Stroke	0	3 (0.4%)	0	1 (0.3%)	4 (0.3%)
Pulmonary embolism	0	4 (0.9%)	0	0	4 (0.3%)
Endocarditis	1 (0.2%)	0	0	0	1 (0.1%)
Non-cardiovascular death	18 (4.2%)	26 (5.8%)	4 (1.2%)	4 (1.2%)	52 (3.4%)
Total	48 (11.1%)	74 (16.6%)	13 (3.9%)	15 (4.6%)	150 (9.8%)

**Figure 2:**
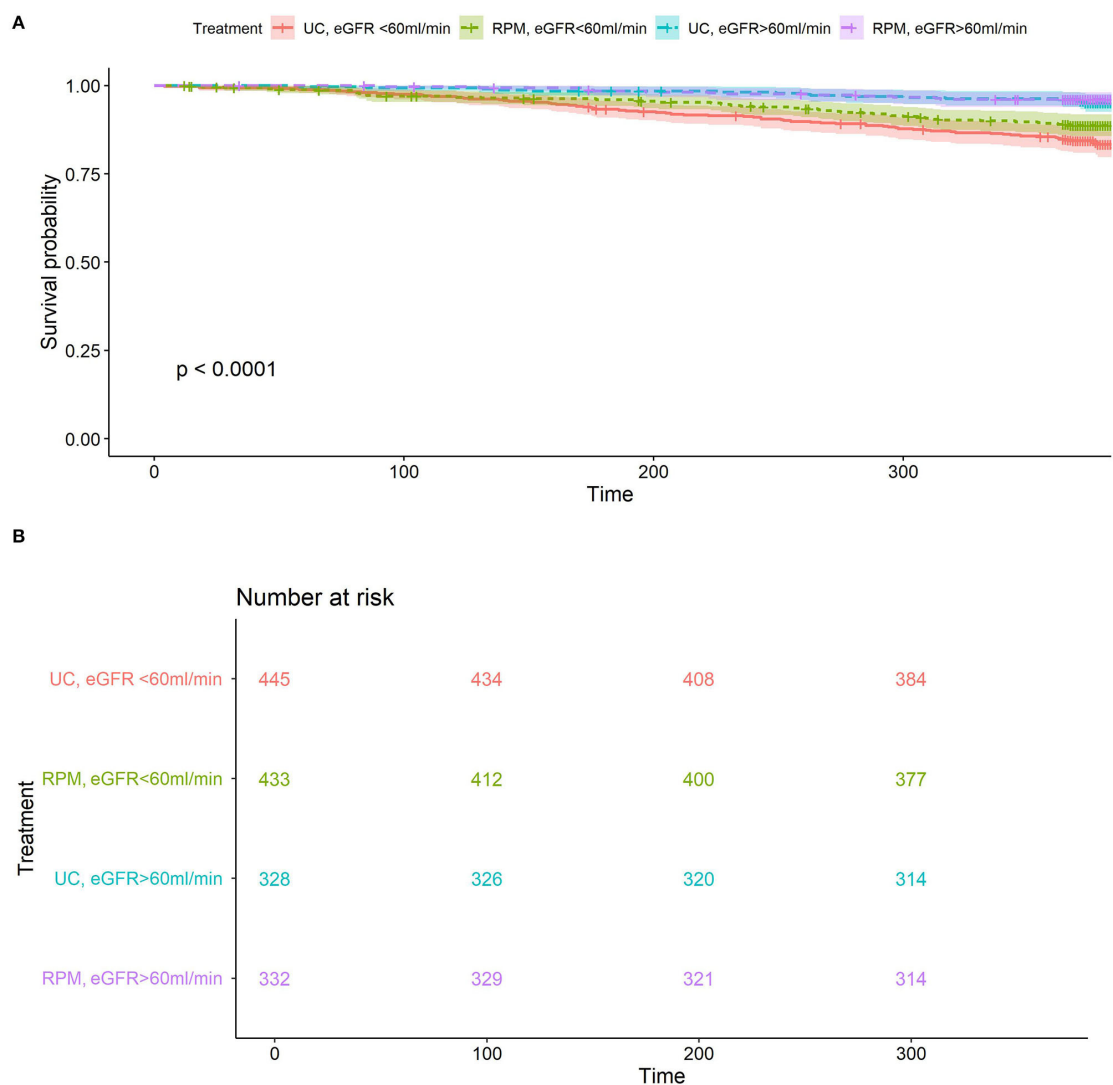
**(A)** All-cause mortality according to randomization arm and renal function. Overall *p* < 0.001, for eGFR subgroup <60 ml/min/1.73 m^2^ *p* = 0.037 RPM (green) vs. UC (red), eGFR subgroup ≥60 ml/min/1.73 m^2^, *p* = 0.7 RPM (purple) vs. UC (blue). **(B)** Number at risk for all-cause mortality according to randomization arm and renal function: eGFR subgroup <60 ml/min/1.73 m^2^, *p* = 0.037 RPM (green) vs. UC (red), eGFR subgroup ≥60 ml/min/1.73 m^2^, *p* = 0.7 RPM (purple) vs. UC (blue).

The cumulative incidence of all-cause mortality by renal function and study group is shown in Figure 2. Patients with eGFR < 60 had a significantly higher 1-year-survival in the RPM than in the UC group (89.4 ± 1.5% vs. 84.6 ± 1.7%, *p* = 0.04), whereas in patients with GFR ≥ 60, 1-year patient survival was similar in both groups (96.0 ± 1.1% vs. 96.3 ± 1.0%, *p* = 0.7).

The percentage of days lost due to unplanned heart failure hospitalization was significantly lower in RPM than in UC [11.4 vs. 18.9%, IRR 0.80 (0.69–0.95), *p* = 0.007] in patients with eGFR ≥ 60. This did not reach significance in patients with eGFR < 60 (23.8 vs. 29.4%, *p* = 0.07).

The recurrent event analyses are shown in Table 5A. Recurrent cardiovascular hospitalizations and all-cause or cardiovascular death, respectively, showed no difference between patients with the renal function above or below 60 ml/min/1.73 m^2^ in both treatment arms.

**Table 5A:** Hospitalization as recurrent event analysis of the cohort according to the treatment arm.

**eGFR**	**<60 ml/min/1.73 m** ^**2**^				**IRR RPM vs. UC**	***p*** **-value**	**≥60 ml/min/1.73 m** ^**2**^				**IRR RPM vs. UC**	***p*** **-value**
	**RPM (*N* = 433)**	**UC (*N* = 445)**			**RPM (*N* = 332)**	**UC (*N* = 328)**						
	**Number of patients with events**	**Weighted average (95% CI)**	**Number of patients with events**	**Weighted average (95% CI)**			**Number of patients with events**	**Weighted average (95% CI)**	**Number of patients with events**	**Weighted average (95% CI)**		
CV hospitalizations (number of patients)	171 (39.5%)		175 (39.3%)			0.9	81 (24.4%)		94 (28.7%)			0.4
CV hospitalizations (sum of hospitalizations)	274	0.70 (0.59, 0.83)	327	0.82 (0.69, 0.96)	0.86 (0.68, 1.08)	0.2	134	0.40 (0.32, 0.50)	153	0.46 (0.37, 0.57)	0.86 (0.64, 1.17)	0.3
CV hospitalizations and all-cause death (number of patients)	177 (40.9%)		194 (43.6%)			0.4	88 (26.5%)		96 (29.3%)		0.86 (0.63, 1.18)	0.4
CV hospitalizations and all-cause death (sum of hospitalizations)	318	0.87 (0.73, 1.04)	398	1.09 (0.92, 1.28)	0.80 (0.63, 1.02)	0.07	147	0.45 (0.36, 0.56)	167	0.52 (0.42, 0.64)	0.86 (0.63, 1.18)	0.4
CV hospitalizations and CV death (number of patients)	175 (40.4%)		186 (41.8%)			0.7	84 (25.3%)		95 (29.0%)			0.5
CV hospitalizations and CV death (sum of hospitalizations)	307	0.83 (0.70, 0.99)	375	0.99 (0.84, 1.17)	0.84 (0.66, 1.07)	0.2	143	0.43 (0.34, 0.54)	164	0.51 (0.41, 0.63)	0.85 (0.62, 1.16)	0.3
HF hospitalizations and all-cause death (number of patients)	119 (27.5%)		156 (35.1%)			0.02	45 (13.6%)		67 (20.4%)			0.02
HF hospitalizations and all-cause death (sum of hospitalizations)	214	0.61 (0.49, 0.76)	304	0.88 (0.72, 1.07)	0.70 (0.52, 0.94)	0.02	66	0.21 (0.15, 0.28)	101	0.32 (0.24, 0.42)	0.64 (0.42, 0.97)	0.04
HF hospitalizations and CV death (number of patients)	112 (25.9%)		145 (32.6%)			0.03	41 (12.3%)		65 (19.8%)			0.01
HF hospitalizations and CV death (sum of hospitalizations)	203	0.58 (0.47, 0.72)	281	0.79 (0.64, 0.97)	0.74 (0.55, 1.00)	0.05	62	0.19 (0.14, 0.26)	98	0.31 (0.24, 0.41)	0.61 (0.40, 0.94)	0.02

*Number of patients mark the frequency of patients affected from hospitalizations, sum of hospitalizations marks the duration of hospitalizations. CI, confidence interval; CV, cardiovascular; eGFR, estimated glomerular filtration rate; HF, heart failure; RPM, remote patient management; UC, usual care; IRR, incident rate ratio. p-value was calculated with a permutation test using individual observation time as weights*.

In patients with eGFR < 60 recurrent heart failure hospitalizations [incidence rate ratio (IRR) 0.74, *p* = 0.05] and the combination of recurrent heart failure hospitalizations and all-cause death (IRR 0.70, *p* = 0.02) were lower in the RPM group than in the UC group, whereas the combination of cardiovascular hospitalizations and all-cause death (IRR 0.80, *p* = 0.07) or heart failure hospitalizations and cardiovascular death (IRR 0.74, *p* = 0.05) were not different.

In patients with eGFR ≥ 60, heart failure hospitalizations (IRR 0.60, *p* = 0.02), the combination of heart failure hospitalizations and all-cause death (IRR 0.64, *p* = 0.04) and heart failure hospitalizations and cardiovascular death (IRR 0.61, *p* = 0.02) were lower in the RPM group than in the UC group.

Changes in lab values and depression scores from baseline to end of observation are shown in Table 5B. A decline of eGFR was observed in RPM and UC in both groups: eGFR ≥ 60 −2.1 vs. −0.8 ml/min/1.73 m^2^, *p* = 0.02 and eGFR < 60: −5 vs. −4 ml/min/1.73 m^2^, *p* = 0.02, respectively. Other biomarkers of renal function remained unchanged on average. NT-proBNP was similar between RPM and UC in patients with eGFR ≥ 60 and <60.

**Table 5B:** Comparison of lab values and MLHFQ from baseline to end of observation (last carried forward) according to the treatment arm.

**eGFR**	**<60 ml/min/1.73 m** ^**2**^			**≥60 ml/min/1.73 m** ^**2**^		
**Group**	**RPM**	**UC**	***p*** **-value**	**RPM**	**UC**	***p*** **-value**
***N***	***N*** ** = 433**	***N*** ** = 445**		***N*** ** = 332**	***N*** ** = 328**	
Creatinine (mg/dl)	0.1 (−0.1 to +0.3)	−0.1 (−0.4 to +0.3)	0.04	0.1 (−0 to +0.2)	0 (−0.1 to +0.2)	0.02
eGFR (ml/min/1.73 m^2^)	−2 (−10 to +3)	−1 (−8 to +5)	0.02	−5 (−17 to +2)	−4 (−12 to +3)	0.02
Potassium (mmol/l)	0 (−0.5 to +0.3)	0 (−0.4 to +0.3)	0.9	0 (−0.4 to +0.2)	0 (−0.3 to +0.4)	0.05
Sodium (mmol/l)	0 (−2 to +2)	0 (−2 to +2)	0.5	0 (−2 to +3)	0 (−2 to +2)	0.1
Hematocrit (%)	0 (−3 to +2)	0 (−2.7 to +2)	0.7	0 (−3 to +2)	0 (−2.7 to +2)	0.9
Hemoglobin (g/dl)	0 (−0.8 to +0.6)	0 (−0.8 to +0.6)	0.8	−0.2 (−1 to +0.6)	0 (−0.9 to +0.6)	0.2
NT-pro BNP (pg/ml)	−17 (−713 to +532)	0 (−577 to +862)	0.3	−93 (−540 to +111)	−134 (−616 to +178)	0.7
MLHFQ			0.06			0.5
Global Score	−1.9 (−11.9 to +5)	0 (−9.9 to +7.4)		−1 (−11 to +7.3)	−1.1 (-13 to +6.1)	

*Data are median and IQR. eGFR, estimated glomerular filtration rate; MLHFQ, Minnesota Living with heart failure questionnaire; RPM, remote patient management; UC, usual care. p-value was calculated using Mann–Whitney U*.

The number of vital signs transmitted was not different in patients with eGFR < 60 and eGFR ≥ 60 in RPM. There were more medication changes in patients with eGFR < 60 than in those with eGFR ≥ 60 (11 vs. 9, *p* = 0.003, Table 6). The distribution of patients receiving diuretics and RAAS medication is given in Supplementary Table 1. There were no differences between RPM and UC in patients with eGFR ≥ 60 and eGFR < 60.

**Table 6:** Measurements in remote patient management and changes in medication in patients with normal and impaired renal function.

**Parameter**	**All**	**eGFR < 60 ml/min/1.73 m^2^**	**eGFR ≥ 60 ml/min/1.73 m^2^**	***p*** **-value**
No of submitted data				
Feeling	344 (310–362)	344 (296–362)	344 (316–362)	0.2
Mean blood pressure	367 (335–391)	367 (327–391)	367 (343–391)	0.3
Systolic blood pressure	367 (335–391)	367 (327–391)	367 (343–391)	0.3
Diastolic blood pressure	367 (335–391)	367 (327–391)	367 (343–391)	0.3
Heart frequency	367 (335–390)	367 (327–391)	367 (343–389)	0.3
Weight	353 (321–372)	352 (312–371)	353 (329–373)	0.1
Changes in medication	3	11 (6–17)	9 (5–14)	0.003

*Numbers are median and IQR. eGFR, estimated glomerular filtration rate, p-value was calculated using Mann–Whitney-U*.

### Cox Regression Analysis for All-Cause Death According to Renal Function

Table 7 shows a cox regression analysis on all-cause death in patients with eGFR < and ≥60 ml/min/1.73 m^2^. An increase of 1 ng/ml NT-pro-BNP showed an 8.1% higher risk of death. An increase of 1 kg/m^2^ in body mass index showed a 3.6% risk reduction. Age at randomization showed a 2.6% higher risk per 1 year. The eGFR subgroup <60 ml/min/1.73 m^2^ showed a risk for death 2.3 times as high as the subgroup eGFR ≥ 60 ml/min/1.73 m^2^. The interaction between sodium at baseline and eGFR was significant and showed a risk reduction of 5% per increase of 1 mmol/l sodium in patients with eGFR < 60 and was not significant in patients with eGFR ≥ 60.

**Table 7:** Cox regression analysis for all-cause death in eGFR subgroups < and ≥ 60 ml/min/1.73 m^2^.

	**HR**	**95% CI for HR**		***p*** **-value**
		**Lower**	**Upper**	
Treatment arm RPM vs. UC	0.775	0.563	1.067	0.1
Potassium at baseline [mmol/l]	0.958	0.741	1.239	0.7
NTproBNP [μg/ml]	1.081	1.058	1.104	<0.001
Age at randomization [years]	1.026	1.005	1.047	0.01
BMI at baseline [kg/m^2^]	0.964	0.932	0.996	0.03
LVEF at baseline ≤ vs. >40%	1.003	0.703	1.429	0.9
Gender [female vs. male]	0.808	0.562	1.161	0.2
eGFR at baseline < vs. ≥60 ml/min/1.73 m^2^	2.270	1.451	3.552	<0.001
Sodium at baseline [mmol/l] ^*^eGFR < *vs*. ≥60 ml/min/1.73 m^2^				0.04
Sodium at baseline [mmol/l], eGFR <60 ml/min/1.73 m^2^	0.941	0.899	0.984	0.01
Sodium at baseline [mmol/l], eGFR ≥60 ml/min/1.73 m^2^	1.074	0.955	1.207	0.2

*The reference group is eGFR ≥ 60 ml/min/1.73 m^2^. CI, confidence interval; BMI, body mass index; eGFR, estimated glomerular filtration rate using CKD-EPI formula; LVEF, left ventricular ejection fraction; NT-pro-BNP, N-terminal prohormone of brain natriuretic peptide; RPM, remote patient management; UC, usual care*.

## Discussion

The present exploratory analyses of prespecified eGFR-subgroups from the TIM-HF2 trial investigate the clinical effects of RPM in an HF population with impaired renal function. Other high-risk groups within the TIM-HF2 study cohort have been investigated and also showed better outcomes in the RPM group (31–33). Thus our paper extends this observation to patients with impaired vs. better renal function. Since patients in the prespecified subgroups had identical risk at baseline, the differences in outcome might be explained by the need for more extensive monitoring of high-risk groups such as patients with poor baseline eGFR.

The strong interrelation between heart and kidney function causing the cardiorenal syndrome has been known for long (3–6). CKD is an important independent risk factor for poor outcomes in patients with cardiovascular disease (7) and impaired renal function is one of the strongest predictors of outcome in HF (10–12, 34). The prevalence of CKD is rising worldwide. There are many biomarkers for prognosis (risk of mortality) and prediction (reaction to treatment) (35). However, in our study that started in 2013 only eGFR was calculated. eGFR has a high prognostic and predictive impact on mortality and disease progression. NT-pro BNP as a main prognostic marker in heart failure patients has limited use in patients with CKD as it is accumulating due to CKD.

Because of the prognostic impact of renal function on heart failure outcomes, the subgroup of patients with the impaired renal function was chosen for this prespecified sub-analysis of the TIM-HF2 trial. Impaired renal function was defined as an eGFR < 60 ml/min/1.73 m^2^ as defined by KDIGO CKD stage III, from which a yearly control is recommended to slow down the further deterioration of renal function (36). The study demonstrates that non-invasive RPM may reduce all-cause mortality and recurrent hospitalizations in this high-risk population. RPM in HF shares key interventions of CKD patient management with optimization of volume status, potassium levels, and blood pressure by medication change (10–12, 34). Furthermore, patients with CKD are prone to cardiovascular comorbidities and suffer from cardiovascular events (37). While the benefit of pharmacological therapies and device therapy has been shown for this subgroup (38–41), the effect of RPM as a new structured approach to prevent unplanned heart failure hospitalizations and death has not yet been studied prospectively (26, 42–48). RPM in the TIM-HF2 trial targets volume management and optimization of cardiac pre- and afterload by a medication change, detection of arrhythmia, patient education, and establishment of collaborative heart failure networks including 24/7 emergency service.

The TIM-HF2 cohort is a well-characterized high-risk HF population with optimal background HF therapy and the use of implantable cardiac devices. The study was initiated in 2013 and groups were stratified with an LVEF cutoff of 45%. In 2016, heart failure guidelines changed introducing a third category (heart failure with mid-range ejection fraction) with LVEF 40 to <50%(27, 49). This class shares features from HFrEF and HFpEF and is underrepresented in current studies (50).

Demographic features and overall event rates are comparable to other recent interventional trials in HF patients (8, 26, 45, 51–53). Patients with TIM-HF2 and impaired renal function had more comorbidities, a higher number of drugs used, and showed higher event rates in terms of hospital admission and mortality, compared to other recent studies (12, 52). The Cox regression analysis shows the known risk factors associated with risk for all causes of death (12, 13). The interaction between sodium at baseline and eGFR was significant in patients with eGFR < 60 and showed a risk reduction in patients with eGFR < 60 for increasing sodium at baseline. The influence of the treatment arm on the overall cohort was not significant. This may be due to underpowering or it may imply that this treatment intervention will be most useful for selected patients with a higher disease burden.

The primary outcome of the percentage of days lost due to unplanned cardiovascular hospitalizations or all-cause death is a relevant endpoint from the patient's perspective. While the main study showed a benefit of using RPM in the primary outcome, we cannot provide further evidence for RPM effects in the subgroups of patients with impaired (eGFR < 60) and better (eGFR ≥ 60) renal function. However, the effect size of RPM was more pronounced in the group of patients with impaired renal function (UC: 24.5 vs. RPM: 34.1 days) than in patients with normal renal function (UC: 9.3 vs. RPM: 10.6 days). As the study was powered to show the effect of RPM on the whole population, it was underpowered to show differences between RPM and UC within these eGFR-subgroups regarding this endpoint.

Our subgroup analysis revealed a 33% reduction (RPM: 11.1% vs. UC: 16.6%) in the secondary endpoint of all-cause mortality in patients with impaired renal function during the 1-year follow-up period, whereas this outcome was not significantly changed in patients with better renal function (eGFR ≥ 60). We conclude that the effect of RPM in the main study is mainly driven by the patients with impaired renal function, a cohort with much higher disease burden and event rates. Therefore, patients with impaired renal function may benefit the most from RPM and may be a suitable target group for receiving this new treatment.

In TIM-HF2 the percentage of days lost due to unplanned cardiovascular hospitalization or death of any cause was lowered by 25% with RPM irrespective of the renal function at baseline. In this sub-analysis, RPM reduced the percentage of days lost due to unplanned hospitalizations due to heart failure in patients with eGFR ≥ 60 significantly by 1.3 days (1.8 vs. 3.1 days). Given the impact of HF on healthcare expenditures this decrease is meaningful in shortening hospital stays. In patients with eGFR < 60 the difference between RPM (5.4 days) and UC (7.5 days) was not significant.

Recurrent events of heart failure hospitalizations have a high impact on individual health, and stress healthcare resources and thus, have become key endpoints in heart failure trials (18, 34, 43, 46). In this study, the incidence, as well as the number of days lost due to recurrent heart failure hospitalizations and all-cause death or cardiovascular death, was lower in RPM than in UC, in both eGFR-groups.

RPM has no influence on the length of the hospital stay, but the lower number of hospitalizations indicates the efficacy of the RPM intervention, even in patients with impaired renal function. Large RCTs with invasive RPM by pulmonary artery sensor measurements showed a similar reduction of first and recurrent HF hospitalization, but not mortality, and did not investigate the effect on patients with impaired renal function (18, 54).

The effect of RPM in patients with impaired renal function might be explained by tighter surveillance and early intervention on evolving problems. Medication changes were more frequent in this vulnerable group illustrating the narrow steady state and need for close monitoring and therapeutic adjustments.

Positive effects of RPM in patients with heart failure on survival, rehospitalization, and length of hospital stay have been reported earlier (19, 20, 24, 25, 55) and have become guideline recommendations for the disease management in HF (27). For the first time, we can demonstrate that RPM is also effective in patients with impaired renal function and may offer additional benefits that lead to lower mortality and less recurrent hospitalizations. Thus, the telemedicine approach can be further refined for patients who might benefit the most from this intervention. The patient group with HF and eGFR < 60 has a high risk for mortality and days admitted to the hospital. All-cause mortality was reduced by 5.5% utilizing RPM reflected into a number needed to treat of 18 to save one life on average. During follow-up, patients in the RPM group showed a more pronounced eGFR-decline compared to UC. This may reflect a more consequent use of drugs like diuretics and RAAS-inhibitors for maintaining a decongested and compensated volume status. A longer follow-up and additional investigation with a focus on medication changes are needed to explore this observation and evaluate renal outcomes.

In conclusion, our study shows that remote patient management may reduce all-cause mortality in the highly vulnerable population of patients with heart failure and CKD. In Germany telemedicine for patients with advanced heart failure has been implemented recently to further reduce mortality (50–53, 55, 56).

## Conclusions

RPM may reduce all-cause mortality and HF hospitalizations in HF-patients with eGFR < 60 ml/min/1.73 m^2^. HF hospitalizations and all-cause death were lower in RPM in both eGFR-subgroups in the recurrent event analysis. A further prospective study for a primary analysis including other renal outcomes and biomarkers is needed for further evaluation.

## Limitations

The intervention was adapted to the German healthcare system and involved collaborations with GPs and cardiologists. The original study was not powered for these subgroup analyses. Data cannot be shared due to National and European Union Laws. A statistical analysis plan can be obtained on request.

The study protocol did not specify renal endpoints (acute kidney injury, dialysis) and renal events were not reported separately. Thus, further analysis, like a competing risk analysis is not possible.

The investigation of lab values in the follow-up was restricted to creatinine in the blood. Data of other renal biomarkers like proteinuria and cystatin c are lacking.

For patients in UC, there was no collection of medication changes, whereas in patients with RPM all changes were trackable. One reason for not collecting UC medication plans was the lack of control mechanisms. The medication data at baseline and final visit at which both study groups were documented were highly variable, and thus, that was beyond the scope of this work.

For patients in UC, there was no collection of medication changes. Thus we only can provide medication data at baseline and final visit for both study groups in Supplementary Table 1 which showed no relevant differences.

## Data Availability Statement

The datasets presented in this article are not readily available because data cannot be shared due to National and European Union Laws. Requests to access the datasets should be directed to marcel.naik@charite.de.

## Ethics Statement

The studies involving human participants were reviewed and approved by the local Ethics Committee at Charite University Medicine Berlin. The patients/participants provided their written informed consent to participate in this study.

## Author Contributions

MN contributed with statistical analysis, data preparation and interpretation, writing and revising the manuscript. KB contributed revising the manuscript. KK contributed to research conception and design, data acquisition, data preparation and interpretation, manuscript drafting, and critical revision of manuscript. EV contributed to statistical analysis, data preparation and interpretation, manuscript drafting, and critical revision of manuscript. MP contributed to statistical analysis, data preparation, interpretation, and revising the manuscript. OA contributed funding and revising the manuscript. SS contributed funding and revising the manuscript. WD contributed revising the manuscript. FK contributed to the research conception and design, data acquisition, data preparation and interpretation, manuscript drafting, critical revision of manuscript, obtaining funding, and supervising the work. SW contributed to data acquisition and critical revision of manuscript. All authors contributed to the article and approved the submitted version.

## Funding

This study was supported by a research grant of the German Federal Ministry of Education and Research (Grant Numbers 13KQ0904A, 13KQ0904B, 13KQ1104A). The TIM-HF2 study was a part of the research and development project Gesundheitsregion der Zukunft—Nordbrandenburg.

## Conflict of Interest

OA and SS are employed by the company Fresenius Medical Care. The remaining authors declare that the research was conducted in the absence of any commercial or financial relationships that could be construed as a potential conflict of interest. The authors declare that this study received funding from German Federal Ministry of Education and Research (grant numbers 13KQ0904A, 13KQ0904B, and 13KQ1104A).

## Publisher's Note

All claims expressed in this article are solely those of the authors and do not necessarily represent those of their affiliated organizations, or those of the publisher, the editors and the reviewers. Any product that may be evaluated in this article, or claim that may be made by its manufacturer, is not guaranteed or endorsed by the publisher.
