# Supplementary material for: Remote Patient Management May Reduce All-Cause Mortality in Patients With Heart-Failure and Renal Impairment
Source: Front Med (Lausanne). 2022 Jul 11;9:917466. doi: 10.3389/fmed.2022.917466 (PMC9309436; doi:10.3389/fmed.2022.917466)
Supplement: Supplementary file 1 [file Table_1.docx]

|  | eGFR<60ml/min | | eGFR>60ml/min | |
| --- | --- | --- | --- | --- |
| Group | RPM | UC | RPM | UC |
| N | 433 | 445 | 332 | 328 |
| ACE Inhibitor | 207 | 193 | 188 | 186 |
| aldosteron antagonist | 222 | 221 | 220 | 185 |
| ARB Inhibitor | 162 | 192 | 117 | 105 |
| beta blocker | 398 | 410 | 307 | 302 |
| loop diuretic | 422 | 431 | 297 | 290 |
| other diuretic | 67 | 80 | 15 | 26 |
| thiazide | 55 | 45 | 44 | 40 |

Supplement Table 1a: Amount of patients at baseline N=1538 and medication for diuretics and RAAS-medication.

|  | eGFR<60ml/min | | eGFR>60ml/min | |
| --- | --- | --- | --- | --- |
| Group | RPM | UC | RPM | UC |
| N | 322 | 309 | 256 | 256 |
| ACE Inhibitor | 197 | 169 | 181 | 176 |
| aldosteron antagonist | 144 | 142 | 153 | 137 |
| ARB Inhibitor | 63 | 68 | 38 | 37 |
| beta blocker | 285 | 274 | 239 | 234 |
| loop diuretic | 311 | 295 | 228 | 219 |
| other diuretic | 43 | 55 | 13 | 21 |
| thiazide | 35 | 27 | 33 | 23 |

Supplement Table 1b: Amount of patients at final visit N=1142 and medication for diuretics and RAAS-medication.
